# Proton pump inhibitors increase the risk of carbapenem-resistant Enterobacteriaceae colonization by facilitating the transfer of antibiotic resistance genes among bacteria in the gut microbiome

**DOI:** 10.1080/19490976.2024.2341635

**Published:** 2024-04-18

**Authors:** Imchang Lee, Jae-Won Jo, Heung-Jeong Woo, Ki Tae Suk, Seung Soon Lee, Bong-Soo Kim

**Affiliations:** a Department of Life Science, Multidisciplinary Genome Institute, Hallym University, Chuncheon, Republic of Korea; b The Korean Institute of Nutrition, Hallym University, Chuncheon, Republic of Korea; c Division of Infectious Diseases, Department of Internal Medicine, Hallym University Dongtan Sacred Heart Hospital, Hallym University College of Medicine, Hwaseong, Republic of Korea; d Division of Gastroenterology and Hepatology, Department of Internal Medicine, Hallym University Chuncheon Sacred Heart Hospital, Hallym University College of Medicine, Chuncheon, Republic of Korea; e Institute for Liver and Digestive Diseases, Hallym University, Chuncheon, Republic of Korea; f Division of Infectious Diseases, Department of Internal Medicine, Hallym University Chuncheon Sacred Heart Hospital, Hallym University College of Medicine, Chuncheon, Republic of Korea

**Keywords:** Carbapenem-resistant enterobacteriaceae, proton pump inhibitors, gut microbiome, antibiotic resistance gene, metagenome-assembled genome

## Abstract

Carbapenem-resistant Enterobacteriaceae (CRE) pose a global health threat; however, there is still limited understanding of the risk factors and underlying mechanisms of CRE colonization in the gut microbiome. We conducted a matched case-control study involving 282 intensive care unit patients to analyze influencing covariates on CRE colonization. Subsequently, their effects on the gut microbiome were analyzed in a subset of 98 patients (47 CRE carriers and 51 non-CRE carriers) using whole metagenome sequences. The concomitant use of proton pump inhibitors (PPIs) and antibiotics was a significant risk factor for CRE colonization. The gut microbiome differed according to PPI administration, even within the CRE and non-CRE groups. Moreover, the transfer of mobile genetic elements (MGEs) harboring carbapenem resistance genes (CRGs) between bacteria was higher in the PPI-treated group than in the PPI-not-treated group among CRE carriers. The concomitant use of PPIs and antibiotics significantly alters the gut microbiome and increases the risk of CRE colonization by facilitating the transfer of CRGs among bacteria of the gut microbiome. Based on these findings, improved stewardship of PPIs as well as antibiotics can provide strategies to reduce the risk of CRE colonization, thereby potentially improving patient prognosis.

## Introduction

Carbapenem-resistant Enterobacteriaceae (CRE) have become an urgent threat in hospitals because of limited treatment options and increased associated mortalities.^1^ Intensive care unit (ICU) patients are exposed to a risk of multi-drug-resistant organism (MDRO) colonization, especially CRE colonization, owing to the use of broad-spectrum antibiotics and various non-antibiotic drugs during hospitalization.^2,3^ However, a comprehensive understanding of the risk factors associated with CRE colonization in the gut microbiome, which is crucial for improving the prognosis of critically ill patients, remains limited.

The gut microbiome plays a crucial role in preventing the proliferation of pathogenic microorganisms, a phenomenon known as colonization resistance.^4,5^ Disruption of this resistance can lead to overgrowth of MDROs such as *Clostridioides difficile*, vancomycin-resistant enterococci, and antibiotic-resistant Enterobacteriaceae in the gut.^5,6^ Understanding these dynamics is paramount, as MDRO expansion in the gut can lead to severe clinical complications, rendering treatment more challenging. Some theories, such as conjugative gene transfer, suggest mechanisms for the transmission of multi-drug resistant genes between bacteria.^7,8^ Horizontal gene transfer (HGT) can play an important role in spreading carbapenem resistance via carbapenemase-bearing plasmids.^9,10^ Although studies indicate that antibiotic therapy contributes to the spread of MDRO, the intricate interactions within the gut microbiome, combined with various confounding factors, make it difficult to pinpoint the exact drivers promoting HGT.^11^

Non-antibiotic pharmaceutical agents can influence the gut microbiome.^12–14^ However, these studies mainly focused on healthy people or patients without antibiotic administration. A recent study reported that proton pump inhibitors (PPIs), the most used acid-suppressant drugs, are associated with an increased risk of MDRO colonization, although it reported only the risk ratio through meta-analyzes without comprehensive analyzes of the gut microbiome.^15–17^ Understanding the influence of non-antibiotic drugs on the gut microbiome in ICU patients receiving broad-spectrum antibiotics is necessary to comprehend CRE colonization in these patients.

To understand risk factors and underlying mechanisms of CRE colonization, we analyzed the influences of various clinical factors obtained from 282 ICU patients. Among these patients, we were able to obtain fecal samples from 98 patients, including 47 with CRE colonization and 51 without CRE colonization during their ICU stay. The gut microbiomes of 98 patients were analyzed using whole metagenome sequencing of available fecal samples. We found that PPIs increased CRE colonization risk by facilitating the transfer of mobile genetic elements (MGEs) harboring carbapenem-resistance genes (CRGs) within the gut microbiome of patients receiving broad-spectrum antibiotics. Our results suggest that not only antimicrobial stewardship but also PPI stewardship can potentially improve clinical prognosis by reducing risk of CRE colonization in ICU patients.

## Results

### PPIs and antibiotics were significant variables associated with CRE colonization in ICU patients

The clinical variables for the 282 enrolled ICU patients (139 with CRE colonization and 143 without CRE colonization during ICU hospitalization) are summarized in Table 1. The average age was 71.5 ± 15.3 years old, and 149 males and 133 females were included. Age, gender, body mass index, and various co-morbidities were not significantly different between the CRE and non-CRE group (*p* > .05). A total of 237 patients (84.04% of enrolled subjects) received continuous antibiotic treatment in the ICU before sample collection. Treatment history of antibiotics and PPIs were significantly different between the CRE and non-CRE group (*p* < .001). Average periods of antibiotics and PPIs treatment were longer in the CRE group than in the non-CRE group (*p* < .001). However, the use of other non-antibiotic drugs such as H2 blockers and statins showed no significant differences between the two groups. *Klebsiella pneumoniae* was the predominant carbapenem-resistant species detected in the CRE group (87.3% of detected CRE species), and *K. pneumoniae* carbapenemase (KPC)-producing CRE was the predominant CRE genotype (86.0% of detected CRE genotypes).

**Table 1.** Summary of clinical data for 282 patients enrolled in this study.

Characteristics	Non-CRE (*n* = 143)	CRE (*n* = 139)	Overall (*n* = 282)	*P* value
Male/Female, n/n	73/70	76/63	149/133	0.1
Age (years), mean ± SD	72.3 ± 15.1	70.8 ± 15.6	71.5 ± 15.3	0.39
Body mass index (kg/m,^2^ mean ± SD	22.3 ± 4	21.8 ± 3.7	22.1 ± 3.9	0.53
Co-morbidities, n (%)				
Atrial fibrillation (Afib)	18(12.58%)	15(10.79%)	33	0.712
Cancer	25(17.48%)	11(7.91%)	36	0.019
Cerebrovascular accident (CVA)	42(29.37%)	41(29.49%)	83	0.999
Chronic kidney disease (CKD; eGFR < 30)	21(14.68%)	26(18.7%)	47	0.425
Congestive heart failure (CHF)	18(12.58%)	11(7.91%)	29	0.240
Coronary artery obstructive disease (CAOD)	10(6.99%)	6(4.31%)	16	0.441
Dementia	28(19.58%)	17(12.23%)	45	0.105
Diabetes (DM)	43(30.06%)	48(34.53%)	91	0.446
Hypertension (HTN)	71(49.65%)	68(48.92%)	139	0.905
Liver cirrhosis (LC)	9(6.29%)	5(3.59%)	14	0.412
Pressure ulcer (Sore)	4(2.79%)	9(6.47%)	13	0.164
Respiratory disease (RD)	21(14.68%)	14(10.07%)	35	0.280
Surgery	38(26.57%)	45(32.37%)	83	0.298
Treatment				
Antibiotics	99(69.23%)	138(99.28%)	237	<0.001
H2 blocker	27(18.88%)	36(25.89%)	63	0.197
Immunosuppressants	7(4.89%)	11(7.91%)	18	0.337
Metformin	15(10.48%)	18(12.94%)	33	0.580
PPI	65(45.45%)	114(82.01%)	179	<0.001
Statin	40(27.97%)	48(34.53%)	88	0.249
Antibiotics treatment period (days), mean ± SD	7.8 ± 10.6	22.6 ± 17.6	15.1 ± 16.2	<0.001
PPI treatment period (days), mean ± SD	10.5 ± 22.6	22.2 ± 22.2	16.3 ± 23.2	<0.001
CRE species detected (n)				
*Citrobacter amalonaticus*	0	1	1	
*Citrobacter freundii*	0	1	1	
*Enterobacter cloacae*	0	6	6	
*Escherichia coli*	0	3	3	
*Klebsiella oxytoca*	0	3	3	
*Klebsiella pneumoniae*	0	124	124	
*Serratia marcescens*	0	4	4	
CRE genotypes detected (n)				
Kpc-CPE	-	123	123	-
Ndm-CPE	-	6	6	-
Non-typeable CPE	-	3	3	
Oxa-CPE	-	1	1	-
Vim/Imp-CPE	-	10	10	-

CRE, Carbapenem-resistant Enterobacteriaceae; SD, Standard deviation; eGFR, Estimated glomerular filtration rate; PPI, Proton pump inhibitors, CPE, Carbapenem-producing Enterobacteriaceae; Kpc, Klebsiella pneumoniae carbapenemase; NDM, New Delhi metallo-β-lactamase; OXA, oxacillinase; VIM, Verona integron metallo-β-lactamase; Imp, imipenemase.

We analyzed the influence of clinical variables on the CRE colonization using odd ratio (OR). Antibiotics exhibited the highest OR value (65.37, 95% CI 8.08–529.01, *p* < .001) and PPIs had the second highest OR value (6.70, 95% CI 3.23–13.87, *p* < .001) among clinical variables based on multivariable analysis (Table S1). Concomitant use of PPIs and antibiotics was detected in most ICU patients. Thus, we evaluated the influence of concomitant use of PPIs and specific antibiotic classes on the CRE colonization (Table S2). The antibiotics used by the enrolled patients were classified into 15 drug classes (Table S3). The ORs for concomitant use of PPIs and antibiotics (7.74, 95% CI 4.47–13.42, *p* < 0.001) were significantly higher than those for administration of antibiotics without PPIs (0.44, 95% CI 0.25–0.77, *p* = .004). The OR values for the administration of carbapenems with PPIs (10.4, 95% CI 4.72–22.94, *p* < .001) and glycopeptides with PPIs (9.48, 95% CI 4.29–20.95, *p* < .001) were relatively higher than those for other antibiotics. These results showed that the concomitant use of PPIs and antibiotics could be associated with CRE colonization.

### The gut microbiome was altered by PPI treatment and CRE colonization

**Figure 1.**
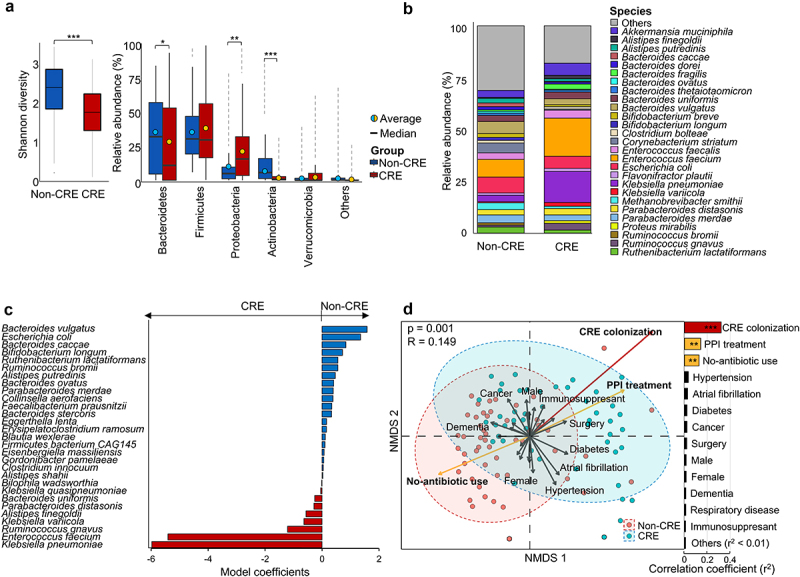
Comparison of gut microbiomes between non-CRE and CRE groups.

The gut microbiome of 98 ICU patients (51 without CRE colonization and 47 with CRE colonization), out of a total of 282 subjects in the study from whom fecal samples could be collected, were analyzed using whole metagenome sequences. The diversity of gut microbiota in the CRE group was lower than that in the non-CRE group (*p* < .001; Figure 1(a)). The relative abundances of Bacteroidetes and Actinobacteria were lower in the CRE group than in the non-CRE group (*p* < .05), whereas Proteobacteria was higher in the CRE group (*p* < .01). Differences in the gut microbiome according to CRE colonization were clear at the species level. The relative abundance of *K. pneumoniae* and *Enterococcus faecium* was higher in the CRE group than in the non-CRE group (*p* < .01; Figure 1(b)). Significantly different species between groups were selected through contributor analysis using the permutational multivariate analysis (PERMANOVA) (*F*-value = 1.6973, degree of freedom = 1). Twenty-nine species were significantly different between groups (*p* < .05; Figure 1(c)). Nine species, including *K. pneumoniae*, *E. faecium*, and *Ruminococcus gnavus*, were discriminating bacteria in the CRE group and 20 species, including *Bacteroides vulgatus* and *Escherichia coli*, contributed to the gut microbiome of the non-CRE group.

(a) The Shannon diversity index and composition of phyla in the gut microbiomes were compared between non-CRE and CRE groups. Phyla with a relative abundance < 1% in each group were combined into the “others”. (b) The compositions of dominant species (>1% in each group) were compared between non-CRE and CRE groups. Species with a relative abundance < 1% in each group were combined into the “others”. (c) Species significantly contributing to the gut microbiomes of CRE and non-CRE groups were identified (*P* < .05). The x-axis indicates significance of ADONIS (PERMANOVA) coefficients. (d) The difference in gut microbiomes between non-CRE and CRE groups were analyzed via a nonmetric multidimensional scaling (NMDS) plot based on the Bray-Curtis distance (*P* = .001). The factors that correlated with the difference in gut microbiomes were determined using the EnvFit model. CRE, carbapenem-resistant Enterobacteriaceae; PPI, proton pump inhibitor; PERMANOVA, permutational multivariate analysis. ***P < .001, **P < .01, *P < .05.

The clinical variables for gut microbiome analyzed 98 patients were compared between CRE carriers and non-CRE carriers. Treatment history and period of antibiotics and PPI were significantly different between groups (*p* < .01; Table S4). Among the clinical variables, PPI treatment was significantly associated with CRE colonization based on multivariable analysis (OR value = 8.93, 95% CI 2.13–37.41, *p* = .003; Table S5). The use of antibiotics demonstrated the high OR value, but it was not statistically significant (14.77, 95% CI 0.68–320.54, *p* = .086). To identify the influence of clinical factors on the variation of the gut microbiome, factors significantly associated with gut microbiome differences between the CRE and non-CRE groups (*p* = .001, *R* = .149 in nonmetric multidimensional scaling [NMDS] plots) were analyzed using the EnvFit model (Figure 1(d)). CRE colonization was the most significant factor distinguishing the gut microbiome between the groups (*p* = .001, r^2^ = 0.2931). PPI treatment was the second most significant factor underlying differences in the gut microbiomes, showing a positive correlation with the gut microbiome of the CRE group (*p* = .003, r^2^ = 0.1295). In addition, no-antibiotic use was a significant factor correlated with the gut microbiome of the non-CRE group (*p* = .007, r^2^ = .1155). Other clinical factors were not associated with the differences between the gut microbiome of each group (*p* > .05).

### Taxonomic and functional features of the gut microbiome differed according to PPI treatment

**Figure 2.**
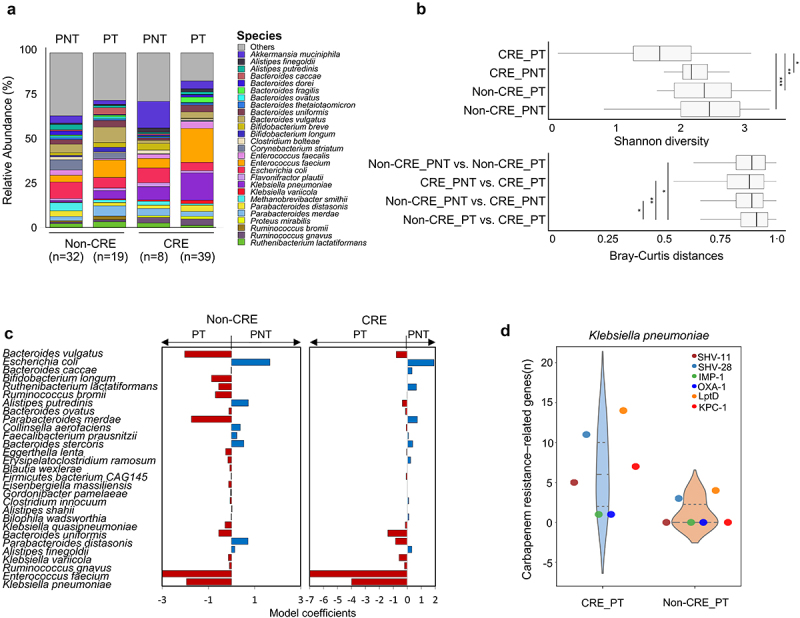
Alterations of the gut microbiome according to PPI treatment.

As PPI treatment was a significant factor in CRE colonization and alteration of the gut microbiome between CRE and non-CRE groups, the influence of PPI treatment on the gut microbiome was analyzed within both the CRE and non-CRE groups. The gut microbiome composition differed according to PPI administration, even within the CRE and the non-CRE group (Figure 2(a)). The relative abundance of *K. pneumoniae* and *E. faecium* increased and that of *E. coli* decreased in PPI-treated patients compared with those in PPI-not-treated patients. Bacterial diversity was lower in the PPI-treated group than in the PPI-not-treated group among patients with and without CRE colonization, and diversity was lowest in the PPI-treated CRE group (*p* < .05; Figure 2(b)). Microbiome differences between PPI-treated CRE and PPI-treated non-CRE groups were greater than those between PPI-not-treated CRE and PPI-not-treated non-CRE groups. Significantly different species based on PPI treatment were detected in both CRE and non-CRE groups (Figure 2(c)). Although CRE bacteria such as *K. pneumoniae* and *E. faecium* were more abundant in the PPI-treated group than in the PPI-not-treated group among both CRE and non-CRE groups, their abundance was higher in the CRE group than in the non-CRE group. The number of carbapenem resistance genes was higher in the metagenome-assembled genome (MAG) of *K. pneumoniae* obtained from the PPI-treated CRE group than in that of *K. pneumoniae* from the PPI-treated non-CRE group (Figure 2(d)).

(a) Compositions of gut microbiomes according to PPI treatment were compared at the species level. (b) The Shannon diversity and inter-variation of the gut microbiome according to PPI treatment were compared. (c) Contributions of 29 species to the gut microbiome according to PPI treatment in non-CRE and CRE groups. The analyzed 29 species were selected as the significant contributors to the microbiomes of non-CRE and CRE groups (as in Figure 1(c)). The x-axis indicates the significance of ADONIS (PERMANOVA) coefficients. (d) The number of carbapenem resistance-related genes in the metagenome assembled genomes (MAGs) of *Klebsiella pneumoniae* was compared between PPI-treated CRE and PPI-treated non-CRE groups. CRE, carbapenem-resistant Enterobacteriaceae; PPI, proton pump inhibitor; PNT, PPI-not-treated; PT, PPI-treated; PERMANOVA, permutational multivariate analysis. ***P < .001, **P < .01, *P < .05.

Although PPI treatment was significantly associated with the gut microbiome in the CRE group, PPI-treated patients were also included in the non-CRE group (*n* = 19) and antibiotics were used in all patients within the PPI-treated group (both PPI-treated non-CRE and PPI-treated CRE groups). This suggests that concomitant use of PPIs and specific antibiotic classes could cause CRE colonization with the gut microbiome alteration. Thus, we analyzed the ORs for CRE colonization according to concomitant use of antibiotics with PPI treatment in 98 patients (Table 2). The concomitant use of PPIs and antibiotics (8.21, 95% CI 3.18–21.22, *p* < .001) was significantly associated with CRE colonization compared to antibiotic use without PPIs (0.29, 95% CI 0.11–0.79, *p* = .015). In particular, six antibiotic classes (glycopeptides, cephalosporins, carbapenems, nitroimidazole, beta-lactam/beta-lactamase inhibitors [BBIs], and fluoroquinolones) with PPI treatment showed significant ORs (*p* < .05). This result is consistent with the previous results for 282 patients (Table S2).

**Table 2.** Comparison of the odds ratio (OR) for the concomitant use of proton pump inhibitors (PPIs) and specific antibiotic classes associated with carbapenem-resistant Enterobacteriaceae (CRE) colonization in the gut microbiome analyzed 98 patients based on multivariable analysis.

Category	Non-CRE (n)	CRE (n)	Odd ratio	95% CI	*P* value
Antibiotics	38	46	14.77	0.68, 320.54	0.086
Antibiotics with PPI	19	39	8.21	3.18, 21.22	<0.001
Antibiotics w/o PPI	19	7	0.29	0.11, 0.79	0.015
BBI	20	30	2.74	1.21, 6.2	0.016
BBI with PPI	10	27	5.54	2.25, 13.63	<0.001
BBI w/o PPI	10	3	0.28	0.07, 1.09	0.066
Carbapenems	8	22	4.73	1.83, 12.19	0.001
Carbapenems with PPI	5	19	6.24	2.1, 18.6	0.001
Carbapenems w/o PPI	3	3	1.09	0.21, 5.69	0.918
Cephalosporins	19	35	4.91	2.06, 11.69	<0.001
Cephalosporins with PPI	11	31	7.05	2.87, 17.32	<0.001
Cephalosporins w/o PPI	8	4	0.5	0.14, 1.78	0.286
Fluoroquinolones	18	25	2.08	0.93, 4.69	0.076
Fluoroquinolones with PPI	8	22	4.73	1.83, 12.19	0.001
Fluoroquinolones w/o PPI	10	3	0.28	0.07, 1.09	0.066
Glycopeptides	4	22	10.34	3.21, 33.35	<0.001
Glycopeptides with PPI	3	18	9.93	2.69, 36.67	0.001
Glycopeptides w/o PPI	1	4	4.65	0.5, 43.21	0.176
Nitroimidazole	5	14	3.9	1.28, 11.89	0.017
Nitroimidazole with PPI	3	13	6.12	1.62, 23.13	0.008
Nitroimidazole w/o PPI	2	1	0.53	0.05, 6.07	0.612
Sulfonamides	2	5	2.92	0.54, 15.82	0.215
Sulfonamides with PPI	2	4	2.28	0.40, 13.07	0.355
Sulfonamides w/o PPI	0	1	NA	NA, NA	NA
Polymyxin E	1	16	25.81	3.26, 204.38	0.002
Polymyxin E with PPI	0	15	NA	NA, NA	NA
Polymyxin E w/o PPI	1	1	1.09	0.07, 17.89	0.953
No antibiotics	13	1	0.06	0.01, 0.51	0.009
No antibiotics with PPI	0	0	NA	NA, NA	NA
No antibiotics w/o PPI	13	1	0.06	0.01, 0.51	0.009

CI, Confidence interval; w/o, without; BBI, Beta-lactam/beta-lactamase inhibitor; NA, Not applicable.

To compare the role of the gut microbiome among the groups, functional features were compared between the CRE and non-CRE groups according to PPI treatment. Twenty KEGG Orthology (KO) terms were selected as significantly different among groups (*p* < .05; Figure S1). The gut microbiome functional features in the PPI-treated CRE group were the most different from those in the PPI-not-treated non-CRE group. The PPI-not-treated non-CRE and PPI-not-treated CRE groups differed only in beta-alanine metabolism. Porphyrin and chlorophyll metabolism, carbon fixation in photosynthetic organisms, and folate biosynthesis differed significantly between the PPI-treated CRE and PPI-not-treated CRE groups.

### CRGs in the gut microbiome were increased by PPI treatment in the CRE group

**Figure 3.**
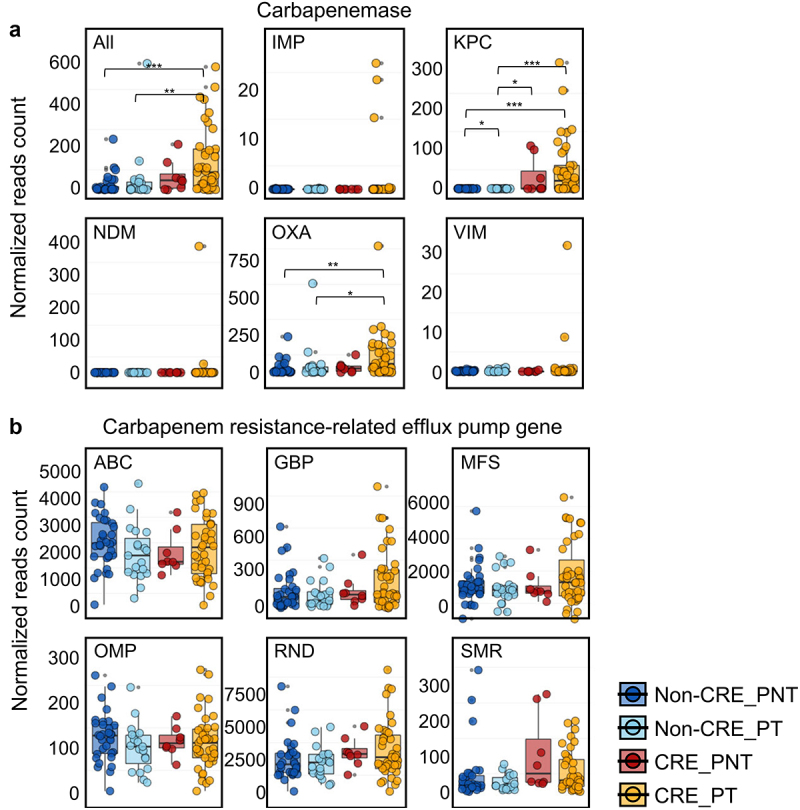
Comparison of normalized counts of carbapenemase genes and carbapenem resistance-related efflux pump genes among groups.

CRE colonization is related to the presence and transfer of CRGs in the gut microbiome.^18,19^ Therefore, we compared relative CRG amounts among the groups according to PPI treatment. Five major carbapenemases (IMP, imipenemase; KPC, *K. pneumoniae* carbapenemase; NDM, New Delhi metallo-β-lactamase; OXA, oxacillinase; VIM, Verona integron metallo-β-lactamase) were compared among the groups. Total levels of all five carbapenemases were higher in the CRE group than in the non-CRE group, and the normalized read counts of carbapenemases were highest in the PPI-treated CRE group (*p* < .01; Figure 3(a)). KPC and OXA levels were significantly higher in the PPI-treated CRE group than in the other groups (*p* < .05). Although no statistical significance was detected, relative KPC and OXA amounts were higher in the PPI-treated CRE group than in the PPI-not-treated CRE group. Efflux pump-related genes, another important carbapenem-resistance factor, were also compared among the groups (Figure 3(b)). The relative amounts of efflux pump-related genes (ABC, ATP-binding cassette; GBP, general bacterial porin; MFS, major facilitator superfamily; OMP, outer membrane porin; RND, resistance-nodulation-cell division; SMR, small multi-drug resistance), obtained from the comprehensive antibiotic resistance database (CARD), were similar among the groups.

(a) Normalized read counts of carbapenemase genes were compared between non-CRE and CRE groups according to PPI treatment. Five major carbapenemase genes were selected for the comparison: IMP, imipenemase; KPC, *K. pneumoniae* carbapenemase; NDM, New Delhi metallo-β-lactamase; OXA, oxacillinase; VIM, verona integron metallo β-lactamase. (b) Distribution of carbapenem resistance-related efflux pump genes. The carbapenem resistance-associated efflux pump genes were retrieved from the CARD database. CRE, carbapenem-resistant Enterobacteriaceae; PPI, proton pump inhibitor; PNT, PPI-not-treated; PT, PPI-treated, CARD, comprehensive antibiotic resistance database; ABC, ATP-binding cassette; GBP, general bacterial porin; MFS, major facilitator superfamily; OMP, outer membrane porin; RND, resistance-nodulation-cell division; SMR, small multi-drug resistance. ***P < .001, **P < .01, *P < .05.

### Antibiotic resistance genes (ARGs) were more frequently transferred among gut microbes in the CRE group according to PPI treatment

For detailed analyzes of ARGs and MGEs in the gut microbiome, we reconstructed MAGs and analyzed them based on the assembled contigs. In total, 1,106 high-quality MAGs were obtained from 98 shotgun metagenomic sequences (737 MAGs with completeness > 80% and 1,035 MAGs with redundancy < 5%; Figure S2) and were classified at the species level (Figure S3).

Based on the MAG analysis, the number of MGEs retrieved from the mobile-OG database was significantly higher in the CRE group than in the non-CRE group, with the highest number detected in the PPI-treated CRE group (*p* < 0.05; Figure S4A). MGEs in the gut microbiome increased according to PPI treatments, even within the non-CRE group (*p* < 0.05). The highest MGE number was detected in the Proteobacteria phylum, and MAGs within Burkholderiaceae and Enterobacteriaceae families contained more MGEs than MAGs in other taxa (Figure S4(b)).

**Figure 4.**
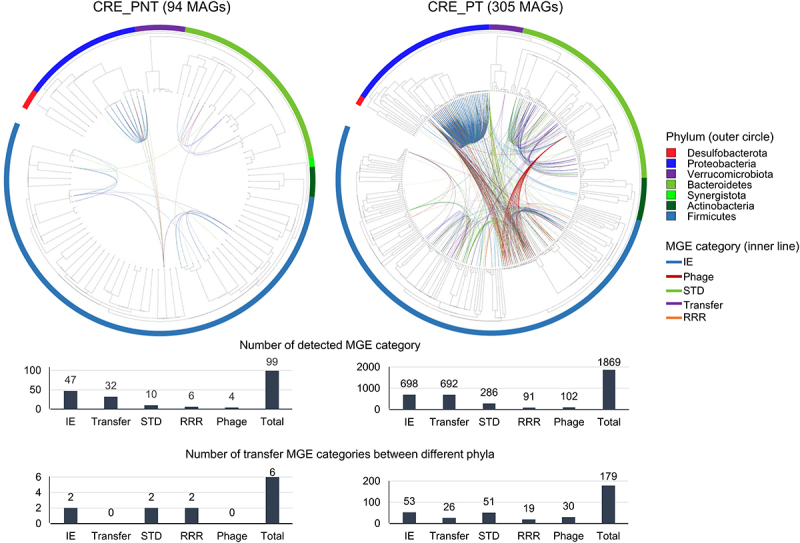
Predicted MGE transfer between bacterial species in CRE-PNT and CRE_PT groups compared using a circular phylogenetic tree based on MAGs.

The high number of MGEs in the gut microbiome of the PPI-treated CRE group could be related to the transfer of ARGs between microbes in the gut. Thus, we analyzed the possible MGE and ARG transfer between gut bacteria in the CRE group. Interestingly, MGE transfer between species was more complex and frequent in the PPI-treated CRE group than in the PPI-not-treated CRE group (Figure 4). The total number of possible MGE transfers was 1,869 in the PPI-treated CRE group and 99 in the PPI-not-treated CRE group. More MGE transfers between different phyla occurred in the PPI-treated CRE group (179) than in the PPI-not-treated CRE group (six). This indicates that extensive transfer potential across phyla was higher in the PPI-treated CRE group than in the PPI-not-treated CRE group.

The unweighted pair group method with arithmetic mean (UPGMA) phylogenetic tree was generated with 94 MAGs in the CRE_PNT group and 305 MAGs in the CRE_PT group. Colors in the outer circle indicate the phyla. Line colors in the inner circle indicate the MGE category. Number of transfer events in each category and number of transfer MGE categories between different phyla were compared. MGE, mobile genetic element; MAG, metagenome assembled genome; PT, proton pump inhibitor (PPI) treated; PNT, PPI-not-treated; IE, integration/excision; STD, stability/transfer/defense; RRR, replication/recombination/repair.

**Figure 5.**
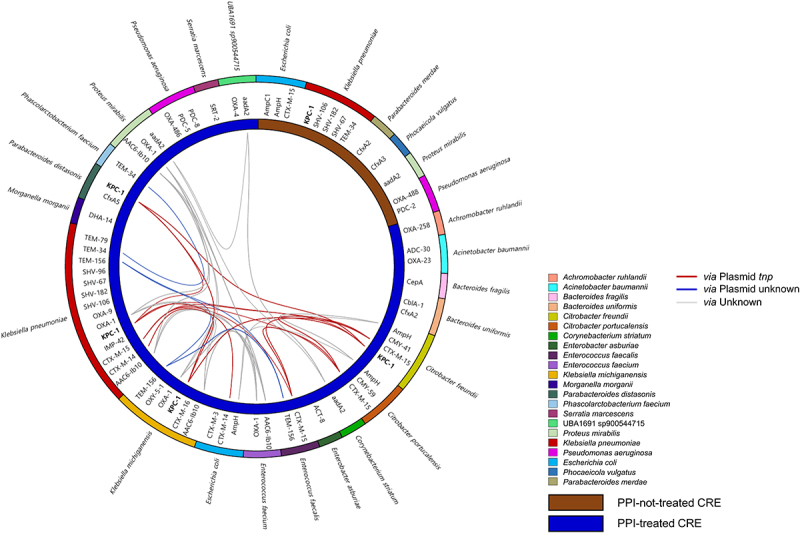
Transfer of carbapenem resistance genes (CRGs) between species in the gut microbiome of CRE carriers according to PPI treatment.

MGE transfer between species was more frequent in the PPI-treated CRE group than in the PPI-not-treated CRE group. We then detailed ARG transfer between species in the gut microbiomes of CRE-carriers to analyze the influence of PPI treatments on carbapenem resistance (Figure 5). CRG-containing species were more diverse in the PPI-treated CRE group (20 species) than in the PPI-not-treated CRE group (six species). Four species (*E. coli*, *K. pneumoniae*, *Proteus mirabilis*, and *Pseudomonas aeruginosa*) were commonly detected as CRG-containing bacteria in both PPI-treated CRE and PPI-not-treated CRE groups. However, the number of detected CRGs was higher in the PPI-treated CRE group than in the PPI-not-treated CRE group. For example, 14 and five CRGs were detected in *K. pneumoniae* in the PPI-treated CRE and PPI-not-treated CRE groups, respectively. Strikingly, the transfer of CRGs, including KPC-1, between species (determined by 100% homology of genes between species) was detected only in the PPI-treated CRE group. Additionally, KPC-1 and CTX-M transfer was predicted mainly based on plasmids harboring the transposase-encoding *tnp* gene.

The transfer of CRGs between species was determined based on 100% nucleotide identify and query coverage > 98% for the paired genes. Colors in the outer circle indicate species, colors in the inner circle indicate PPI treatment, and lines in the circle indicate the processes of transfer. A red line indicates that transfer can occur via a plasmid harboring the *tnp* gene, a blue line indicates that transfer can occur via a plasmid not harboring the *tnp* gene, and a gray line indicates that transfer can occur through an unknown mechanism. CRE, carbapenem-resistant Enterobacteriaceae; PPI, proton pump inhibitor.

## Discussion

In this study, the CRE colonization risk factors and their influence on the gut microbiome were analyzed in patients with and without CRE colonization admitted to the ICU. Although PPI treatment as well as antibiotic administration were both significant factors associated with CRE colonization, the sole use of PPIs was not correlated to CRE colonization. Concomitant use of PPIs and antibiotics, including carbapenems, was significantly associated with CRE colonization in ICU patients. The PPI treatment also influenced the gut microbiome, including ARG transfer between bacteria. The relative abundances of *K. pneumoniae* and *E. faecium* increased with PPI treatment, even within the CRE group. The relative amounts of MGEs and ARGs were higher in the PPI-treated CRE group than in the PPI-not-treated CRE group, and their transfer was more complex and frequent in the PPI-treated CRE group. The transfer of CRGs, including KPC, between species was detected only in the PPI-treated CRE group. Therefore, concomitant use of PPIs and antibiotics increases the risk of CRE colonization through ARG transfer in the gut microbiome.

Several studies have reported differences in the gut microbiome composition between CRE and non-CRE groups.^20–22^ Furthermore, PPI treatment has been reported as a risk factor for Enterobacteriaceae infection and CRE colonization.^2,16,23,24^ However, previous studies have analyzed the gut microbiota composition based on 16S amplicons and focused on the risk of PPI treatment for CRE colonization using statistical analysis. Our study revealed that the risk of CRE colonization is increased by concomitant PPI and antibiotic use via ORs analysis, thereby detailing the alterations of the gut microbiome and CRG transfer among species based on whole metagenome analyzes. PPI treatment without antibiotics was not associated with CRE colonization. These results suggest that the concomitant use of PPIs and antibiotics increases the risk of CRE colonization in ICU patients.

The proportion of *K. pneumoniae* and *E. faecium* increased, whereas that of *E. coli* decreased according to PPI treatment in both CRE and non-CRE groups. This indicates that PPIs could alter the gut microbiome and reduce colonization resistance through antibiotic-like side effects.^12,13^ PPIs increase the abundance of oral and upper gastrointestinal microbiome and decrease microbial diversity due to a decrease in the pH barrier between the upper and lower gastrointestinal tracts.^25^ PPI-induced alterations of the gut microbiome could be related to changes in microbiome functions. Porphyrin and chlorophyll metabolism, carbon fixation in photosynthetic organisms, and folate biosynthesis were decreased in the PPI-treated CRE group. Porphyrin and folate are related to vitamin B12 synthesis,^26^ and the use of PPI has been correlated with vitamin B12 deficiency.^27^ Therefore, species contributing to porphyrin and folic acid metabolism were reduced, which could result in vitamin B12 deficiency caused by PPI treatment in the CRE group.

Relative CRG and MGE levels in the gut microbiome were increased according to PPI treatment in the CRE group. Furthermore, MGE transfer between different phyla was more frequent in the PPI-treated CRE group than in the PPI-not-treated CRE group. This indicates that extensive CRG spread in the gut microbiome could occur upon PPI treatment in CRE-carriers. Detailed analysis through MAG reconstruction showed that the transfer of CRGs, including KPC-1, frequently occurred in the PPI-treated CRE group. *K. pneumoniae* in the PPI-treated CRE group had more diverse CRGs than that in the PPI-not-treated CRE group. Dynamic KPC gene transfer was detected among species, including *K. pneumoniae*, *Klebsiella michiganensis*, *Citrobacter freundii*, and *Parabacteroides distasonis* in the PPI-treated CRE group. CRG transfer between gut microbes in the PPI-treated CRE group was detected mainly by plasmids, as previously reported.^28^ These results are evidence of the dynamic transfer of CRGs in the gut microbiome of CRE-carriers attributed to concomitant PPI and antibiotic use. Although we tested the KPC gene transfer from *K. pneumoniae* to *C. freundii* upon PPI treatment or co-treatment with PPIs and antibiotics based on *in vitro* culture, transfer was not detected. This suggests that dynamic CRG transfer could not be caused by a single bacterium but could be caused by an altered microbiome upon concomitant PPI and antibiotic use at a complex microbiome level.

The influence of PPIs on the microbiome has been reported in several studies.^14,24,25^ The complex interaction between the gut microbiome and the human body may be influenced by the short half-life of PPIs and their prolonged duration of action due to irreversible binding.^29^ The use of PPIs as acid-suppressor can lead to abnormal small intestine bacterial overgrowth,^30^ disrupting the gut microbiome and facilitating gene transfer to adapt to changing gut environment.^31,32^ The combined effects of small intestine bacterial overgrowth and antibiotic-induced alteration of the microbiome ecosystem can enhance gene transfer, which is a widespread mechanism in microbial evolution.^31–33^ However, experimentally elucidating these hypotheses poses a significant challenge due to the complex interaction between the gut microbiome and human host. Further studies are necessary to clarify our results using other techniques, including metatranscriptomics and model systems. Nevertheless, our study is significant because it provides evidence that concomitant PPI and antibiotic use can increase CRE colonization risk through gut microbiome alteration.

Our study had several limitations. First, the gut microbiome was not analyzed using longitudinal data. Longitudinal studies, including tracking changes in the gut microbiome, metabolites, and ARGs, would be helpful in explaining the risk factors and mechanisms of CRE colonization. Second, relatively few subjects were used in the gut microbiome analyzes. Obtaining written informed consent for fecal sample collection from ICU patients was challenging in this study due to the nature of the samples. In addition, although patients ate a standardized hospital diet in acute care hospitals, it was difficult to evaluate dietary effects on the gut microbiome. These limitations will be improved in further longitudinal studies with expanded subject numbers.

Notwithstanding these limitations, to the best of our knowledge, this study is pioneering in showing that concomitant use of PPIs and broad-spectrum antibiotics amplifies the risk of CRE colonization by promoting ARG transfer. Up to 70% of PPI prescriptions are used unnecessarily long-term without clear indications.^34^ Additionally, as a bundle approach to prevent ventilator-associated pneumonia, stress ulcer prophylaxis using PPIs is widely performed in the ICU.^35–37^ Considering the importance of CRE as an urgent antibiotic threat and our findings of an increased CRE colonization risk with the concomitant use of PPIs and broad-spectrum antibiotics, antibiotic and PPI stewardship are needed. Our study provides important value regarding the need for PPI stewardship to prevent the colonization of MDROs, especially CRE.

In conclusion, this study is the first to delineate the intricate interplay between CRE colonization and the gut microbiome, highlighting the significant influence of concomitant use of PPIs and antibiotics in increasing CRE colonization risk. The complex and frequent transfer of MEGs harboring CRGs between species in the gut microbiome occurred under PPI treatment within the CRE group. This study enhances our comprehension of non-antibiotic drug effects on CRE colonization through microbiome analysis at the species level, ARG analysis using the whole metagenome, and MGEs analysis by reconstruction of MAGs. Additionally, this study provides valuable insights for future research directions and clinical approaches to mitigate the risk of CRE colonization.

## Materials and methods

### Study design, study subjects, and sample collection

To assess risk factors for CRE colonization in ICU patients, a case-control study was conducted by recruiting patients with CRE colonization and matching them with patients without CRE colonization based on gender and age. Subjects were prospectively enrolled at the ICU of Hallym University Chuncheon Sacred Heart Hospital, an acute care hospital in the Republic of Korea, between September 2019 and February 2021. Clinical data and stool samples were collected from non-CRE-carriers (based on negative CRE screening results and/or clinical cultures) and CRE-carriers (based on positive CRE screening results and/or clinical cultures) during hospitalization.

Clinical data was collected from 282 enrolled ICU patients (139 with CRE colonization and 143 without CRE colonization during hospitalization) using their medical records. Drug-use history was examined for the following drugs administered within three months before stool collection: acid suppressants (PPIs and H2 blockers) and systemic antibiotics (penicillin, BBIs, cephalosporins, carbapenems, fluoroquinolones, glycopeptides, nitroimidazole, lincosamide, macrolides, polymyxin E, sulfonamides, tetracyclines, and aminoglycosides) administered for more than 72 h. Immunosuppressive therapy was defined as corticosteroid use at ≥20 mg of prednisolone or equivalent for more than three weeks or ≥10 mg/day for at least three months, chemotherapy, or other recognized T-cell immunosuppressants. The history of statin and metformin use was also recorded.

Whole metagenome sequences were analyzed using available fecal samples from 98 patients (47 CRE-carriers and 51 non-CRE carriers), who provided written informed consent for fecal sample collection among 282 ICU patients. This allowed us to evaluate the impact of risk factors for CRE colonization on the gut microbiome, as well as their potential mechanisms. To analyze the gut microbiome, fecal samples were stored at − 80°C until DNA extraction.

The study protocol was approved by the Institutional Review Board at Hallym University Chuncheon Sacred Heart Hospital (IRB no. 2019-05-012). Written informed consent for study participation was obtained from all patients and fecal samples for gut microbiome analysis were collected only from patients who provided consent for sample collection. This study complied with the Declaration of Helsinki.^38^

### Culture and polymerase chain reaction (PCR) for CRE detection

Specimens were collected from the patients during the study period. Culture assays and PCR for carbapenemase-producing CRE detection were performed as described previously.^39^ Primary CRE screening of rectal swabs was conducted using both the CHROMagar^TM^ KPC (CHROMagar, Paris, France) and BD MAX^TM^ CRE assay (Becton Dickinson, Heidelberg, Germany). Blood agar (Becton-Dickinson, Sparks, MD, USA) and MacConkey agar (Becton-Dickinson) were used to cultivate other clinical specimens, such as sputum, urine, and blood. Cultivation was performed at 36.5°C for 16–24 h. Taxonomic identification and antibiotic susceptibility tests, including carbapenem, for isolates were performed using the VITEK® 2 system (bioMerieux, Marcy I’Etoile, France) according to the Clinical and Laboratory Standards Institute M100S guidelines. The RAPIDEC® CARBA NP (bioMerieux, Marcy I’Etoile, France) was used to identify carbapenemase production in each isolate. To validate carbapenemase production in isolates, target genes of *bla*_KPC_, *bla*_NDM_, *bla*_IMP_, *bla*_VIM_, and *bla*_OXA-48_ were detected by multiplex PCR.

### DNA extraction and whole metagenome sequencing

Metagenomic DNA was extracted from stool samples using the RNeasy PowerMicrobiome Kit (Qiagen, Hilden, Germany). Quantification of the extracted DNA was performed using the BioPhotometer D30 & μCuvette G1.0 (Eppendorf, Hamburg, Germany). The extracted DNA was fragmented using NEBNext dsDNA Fragmentase (New England Biolabs, Ipswich, MA, USA). Fragmented DNA was used to prepare metagenomic libraries using a Swift 2S Turbo DNA Library Kit (Swift Biosciences, Ann Arbor, MI, USA) according to the manufacturer’s instructions. Indexing of the adapter-attached DNA was performed using the Swift Combinatorial Dual Indexing Primer Kit (Swift Biosciences) on an Illumina platform. The size of the libraries was confirmed using a Bioanalyzer 2100 (Agilent Technologies, Santa Clara, CA, USA), and the concentration was measured using a PicoGreen dsDNA Assay kit (Invitrogen, Carlsbad, CA, USA). The concentration of the libraries was calculated again through quantitative PCR using a TaKaRa PCR Thermal Cycler Dice Real Time System III (TaKaRa Bio, Inc., Shiga, Japan) for pooling. Equimolar concentrations of each library were pooled and sequenced using the Illumina Nova-Seq 6000 platform (250 bp paired-ends).

### Analysis of whole metagenome sequences

The removal of adapters and quality filtering of sequence data were performed using Trimmomatic.^40^ Merging of the paired-end sequencing data was performed using PEAR v0.9.11.^41^ To remove the contaminated human genes from sequences, BBMap (http://sourceforge.net/project/bbmap) was used with a reference human genome. Taxonomic annotation and functional gene profile analysis were performed using the HMP Unified Metabolic Analysis Network (HUMAnN v3.0).^42^ Functional features of the resultant UniRef90 IDs were converted to Kyoto Encyclopedia of Genes and Genomes (KEGG) Orthology (KO) terms. The normalization of read counts for each feature was performed using the cumulative sum scaling method.^43^

### Reconstruction of MAGs

For reconstruction of the MAG, three binning tools (CONCOCT, MaxBin 2.0, and metaBAT 2) were used for initial binning.^44–46^ The assembly was performed using MEGAHIT v1.2.9 with default options.^47^ The Bin-refinement module within metaWRAP programs was used for consolidation into a single bin within each bin set.^48^ The quality check of each bin, including the completeness and redundancy of the bin, was performed using CheckM v1.1.3.^49^ MAGs with completeness > 50% and redundancy (contamination) <10% were used for further analyzes. Taxonomical assignment was performed using GTDB-Tk v1.7.0.^50^ OrthoANI was used to calculate the average nucleotide identity distance between MAGs, and genomic trees were obtained using iTOL v5.14.^51,52^

### Analysis of MGEs and ARGs

The MGEs in each MAG were predicted based on the mobile-OG database.^53^ Five categories of MGEs, including phages, replication/recombination/repair (RRR), stability/transfer/defense (STD), transfer, and integration/excision (IE), were detected in our results. Phage-associated biological processes included structural proteins, viral genome packaging/lysis/lysogenic-related machinery and regulatory proteins, and virus-encoded cofactors for integration and excision. Genetic elements in RRR are related to proteins mediating the replication, recombination, or repair of MGEs, including plasmid/phage replication initiation/regulation proteins, proteins for the repair system, and homologous recombination protein systems. STD is related to the stability or defense of an element from the host machinery or the machinery of other elements. These include restriction-modification systems, clustered regularly interspaced short palindromic repeats (CRISPR) and anti-CRISPR systemic proteins, and several antiphage system components. Transfer refers to the proteins mediating the transfer of elements between organisms, including binding machineries such as the type IV secretion system, Ftsk/SpoEIII domain-containing proteins, and natural transformation-associated proteins. IE is related to proteins that control, mediate, or assist in site-specific recombination of MGEs, including tyrosine recombinases, transposases, and transcriptional regulator/cofactor-related proteins.^53^ MGEs in each MAG were detected using DIAMOND v0.8.22^54^ by the criteria of amino acid sequence similarity > 50% and a query coverage > 80%.

ARGs in each MAG were detected using the CARD and the Resistance Gene Identifier (CARD-RGI) program with the ‘Perfect’ criterion.^55^ The gene transfer between bacteria was determined using USEARCH v8 by 100% nucleotide identity and query coverage > 98% for the paired genes detected on two different MAGs, setting a higher standard for precision compared to prior studies.^56,57^ The location of detected genes in the MGEs was predicted using the mobile-OG database. Genes located on the plasmid in the resultants were determined to have been transferred through the plasmid. The gene name of MGE in the plasmid was identified using information from the plasmid RefSeq within the mobile-OG database. Genes with unidentified names and uncertain locations were labeled as ‘unknown’ for accurate analysis.

### Statistical analysis

Categorical variables were compared between CRE carriers and non-CRE carriers using a two-tailed Chi-squared test or Fisher’s exact test, and continuous variables were compared using the two-tailed Student’s t-test or Mann-Whitney U test, as appropriate. The OR based on the logistic regression was used to analyze the association between clinical variables and CRE colonization. The logistic regression analysis was performed using the ‘*Logit*’ function in the statsmodels Python library. The constant term (intercept) was integrated into the model with the ‘add_constant’ method to determine the potential absence of all predictors in certain scenarios. The calculation of OR was used for the resultant coefficients of each predictor. The significance of each predictor within the model was determined using the corresponding *p* value. The confidence intervals (CIs) for the log odds with subsequent exponentiation were used to determine the 95% CIs. The calculation of OR was based on multivariate analysis.

The differences in the gut microbiome among samples were analyzed using a NMDS plot based on the Bray – Curtis dissimilarity matrix.^58,59^ For visualization, the monoMDS function implemented in the R vegan package was used, and 100 iterations and the lowest stress were used for multidimensional scaling (MDS).^60^ A two-tailed Mann – Whitney U test, independent samples t-test, and analysis of similarity were used to calculate the significance of differences between samples. To calculate the α-diversity within each sample and β-diversity between the samples, the Shannon diversity index and Bray-Curtis dissimilarity were used. To calculate the model coefficient for the separation, PERMANOVA was used in the R vegan package (v2.5–7).^60^

To identify factors influencing the gut microbiomes among groups, the effect size and significance of each covariate in the variation of the microbiome were determined using the ‘EnvFit’ function in the R vegan package. The significance was determined using 999 permutations. The fitting to the ordination of covariates is shown in the NMDS plots. Statistical significance was set at *p* < .05.

## Supplementary Material

Supplementary materials_revision.docx

## Data Availability

The sequencing data obtained from this study are available in the EMBL SRA database under the study number PRJEB57404 (http://www.ebi.ac.uk/ena/data/view/PRJEB57404) and the sample numbers ERS14237031 to ERS14237128.
