# Supplementary material for: Proton pump inhibitors increase the risk of carbapenem-resistant Enterobacteriaceae colonization by facilitating the transfer of antibiotic resistance genes among bacteria in the gut microbiome
Source: Gut Microbes. 2024 Apr 18;16(1):2341635. doi: 10.1080/19490976.2024.2341635 (PMC11028007; doi:10.1080/19490976.2024.2341635)

Supplementary materials

Proton pump inhibitors increase the risk of carbapenem-resistant Enterobacteriaceae colonization by facilitating the transfer of antibiotic resistance genes among bacteria in the gut microbiome

Imchang Lee^1,^ ^2^, Jae-Won Jo^1,^ ^2^, Heung-Jeong Woo^3^, Ki Tae Suk^4,^ ^5^, Seung Soon Lee^6*^, Bong- Soo Kim^1,^ ^2*^

^1^Department of Life Science, Multidisciplinary Genome Institute, Hallym University, Chuncheon, Gangwon-do, Republic of Korea

^2^The Korean Institute of Nutrition, Hallym University, Chuncheon, Gangwon-do, Republic of Korea

^3^Division of Infectious Diseases, Department of Internal Medicine, Hallym University Dongtan Sacred Heart Hospital, Hallym University College of Medicine, Hwaseong-si, Gyeonggi-do, Republic of Korea

^4^Division of Gastroenterology and Hepatology, Department of Internal Medicine, Hallym University Chuncheon Sacred Heart Hospital, Hallym University College of Medicine, Chuncheon, Gangwon-do, Republic of Korea

^5^Institute for Liver and Digestive Diseases, Hallym University, Chuncheon, Gangwon-do, Republic of Korea

^6^Division of Infectious Diseases, Department of Internal Medicine, Hallym University Chuncheon Sacred Heart Hospital, Hallym University College of Medicine, Chuncheon, Gangwon-do, Republic of Korea

**Table S1.** Odds ratios for clinical variables associated with carbapenem-resistant Enterobacteriaceae (CRE) colonization in 282 enrolled patients.

| **Characteristics Non-CRE CRE** | | | **B**  **Odd ratio** | **ivariable analysi**  **95% CI** | **s**  ***P* value** | **Mu**  **Odd ratio** | **ltivariable analys**  **95% CI** | **is**  ***P* value** |
| --- | --- | --- | --- | --- | --- | --- | --- | --- |
| Female | 73 | 76 | 1.15 | 0.72, 1.83 | 0.541 |  |  |  |
| Male | 70 | 63 | 0.86 | 0.53, 1.37 | 0.541 |  |  |  |
| Co-morbidities |  |  |  |  |  |  |  |  |
| Atrial fibrillation | 18 | 15 | 0.84 | 0.4, 1.74 | 0.639 |  |  |  |
| Cancer | 25 | 11 | 0.4 | 0.18, 0.84 | 0.016 |  |  |  |
| Cerebrovascular accident | 42 | 41 | 1.0 | 0.59, 1.66 | 1.0 |  |  |  |
| Chronic kidney disease  (eGFR<30) | 21 | 26 | 1.33 | 0.7, 2.49 | 0.365 |  |  |  |
| Congestive heart failure | 18 | 11 | 0.59 | 0.26, 1.29 | 0.196 |  |  |  |
| Coronary artery obstructive disease | 10 | 6 | 0.6 | 0.21, 1.69 | 0.331 | 0.25 | 0.07, 0.92 | 0.037 |
| Dementia | 28 | 17 | 0.57 | 0.29, 1.09 | 0.092 | 0.39 | 0.16, 0.93 | 0.034 |
| Diabetes | 43 | 48 | 1.22 | 0.73, 2.01 | 0.422 |  |  |  |
| Hypertension | 71 | 68 | 0.97 | 0.6, 1.54 | 0.902 |  |  |  |
| Liver cirrhosis | 9 | 5 | 0.55 | 0.17, 1.68 | 0.297 |  |  |  |
| Pressure ulcer (Sore) | 4 | 9 | 2.4 | 0.72, 7.98 | 0.141 |  |  |  |
| Respiratory disease | 21 | 14 | 0.65 | 0.31, 1.33 | 0.24 |  |  |  |
| Surgery | 38 | 45 | 1.32 | 0.78, 2.2 | 0.285 |  |  |  |
| Treatment |  |  |  |  |  |  |  |  |
| Antibiotics | 99 | 138 | 61.33 | 8.3, 452.66 | <0.001 | 65.37 | 8.08, 529.01 | <0.001 |
| H2 blocker | 27 | 36 | 1.5 | 0.85, 2.63 | 0.157 | 2.30 | 1.07, 4.95 | 0.033 |
| Immunosuppressants | 7 | 11 | 1.66 | 0.62, 4.41 | 0.3 |  |  |  |
| Metformin | 15 | 18 | 1.26 | 0.6, 2.61 | 0.52 |  |  |  |
| PPI | 65 | 114 | 5.47 | 3.17, 9.42 | <0.001 | 6.70 | 3.23, 13.87 | <0.001 |
| Statin | 40 | 48 | 1.35 | 0.81, 2.23 | 0.234 |  |  |  |

CI, confidence interval; eGFR, Estimated glomerular filtration rate; PPI, Proton pump inhibitors.

**Table S2.** Comparison of the odds ratio (OR) for the concomitant use of proton pump inhibitors (PPIs) and specific antibiotic classes associated with carbapenem-resistant Enterobacteriaceae (CRE) colonization based on multivariable analysis.

| **Category** | **Non-CRE**  **(n)** | **CRE (n)** | **Odd ratio** | **95% CI^a^** | ***P* value** |
| --- | --- | --- | --- | --- | --- |
| Antibiotics | 99 | 138 | 65.37 | 8.08, 529.01 | <0.001 |
| Antibiotics with PPI | 53 | 114 | 7.74 | 4.47, 13.42 | <0.001 |
| Antibiotics w/o^b^ PPI | 46 | 24 | 0.44 | 0.25, 0.77 | 0.004 |
| BBI | 54 | 93 | 3.33 | 2.04, 5.44 | <0.001 |
| BBI with PPI | 32 | 77 | 4.31 | 2.57, 7.22 | <0.001 |
| BBI w/o PPI | 22 | 16 | 0.72 | 0.36, 1.43 | 0.342 |
| Carbapenems | 16 | 63 | 6.58 | 3.55, 12.21 | <0.001 |
| Carbapenems with PPI | 8 | 53 | 10.4 | 4.72, 22.94 | <0.001 |
| Carbapenems w/o PPI | 8 | 10 | 1.31 | 0.5, 3.42 | 0.584 |
| Cephalosporins | 56 | 100 | 3.98 | 2.42, 6.57 | <0.001 |
| Cephalosporins with PPI | 29 | 83 | 5.83 | 3.43, 9.9 | <0.001 |
| Cephalosporins w/o PPI | 27 | 17 | 0.6 | 0.31, 1.16 | 0.126 |
| Fluoroquinolones | 49 | 71 | 2 | 1.24, 3.23 | 0.005 |
| Fluoroquinolones with PPI | 24 | 57 | 3.45 | 1.98, 6 | <0.001 |
| Fluoroquinolones w/o PPI | 25 | 14 | 0.65 | 0.34, 1.23 | 0.185 |
| Glycopeptides | 15 | 60 | 6.48 | 3.45, 12.18 | <0.001 |
| Glycopeptides with PPI | 8 | 50 | 9.48 | 4.29, 20.95 | <0.001 |
| Glycopeptides w/o PPI | 7 | 10 | 1.51 | 0.56, 4.08 | 0.42 |
| Nitroimidazole | 13 | 30 | 2.75 | 1.37, 5.53 | 0.005 |
| Nitroimidazole with PPI | 7 | 27 | 4.68 | 1.97, 11.16 | <0.001 |
| Nitroimidazole w/o PPI | 6 | 3 | 0.5 | 0.12, 2.05 | 0.339 |
| Sulfonamides | 3 | 14 | 5.23 | 1.47, 18.62 | 0.011 |
| Sulfonamides with PPI | 2 | 13 | 7.27 | 1.61, 32.85 | 0.01 |
| Sulfonamides w/o PPI | 1 | 1 | 1.03 | 0.06, 16.61 | 0.984 |
| Polymyxin E | 2 | 28 | 17.78 | 4.15, 76.25 | <0.001 |
| Polymyxin E with PPI | 0 | 23 | NA | NA, NA | NA |
| Polymyxin E w/o PPI | 2 | 5 | 2.63 | 0.50, 13.79 | 0.253 |
| No antibiotics | 44 | 1 | 0.02 | 0, 0.12 | <0.001 |
| No antibiotics with PPI | 12 | 0 | NA | NA, NA | NA |
| No antibiotics w/o PPI | 32 | 1 | 0.03 | 0, 0.19 | <0.001 |

CI, Confidence interval; w/o, without; PPI, Proton pump inhibitors; BBI, Beta-lactam/beta-lactamase inhibitor; NA, Not applicable.

**Table S3.** Drug classes of antibiotics used in this study.

**Drug class Antibiotics**

Aminoglycosides Amikacin

Ansamycins Rifamycin

Beta-lactam/Beta-lactamase Inhibitors (BBI)

Piperacillin/Tazobactam Amoxicillin/Clavulanate Cefoperazone/Sulbactam Ampicillin/Sulbactam

Carbapenems Imipenem/Cilastatin

Meropenem Ertapenem

Cephalosporins Cefepime

Cefotaxime Ceftriaxone Cephalexin Ceftazidime Cefroxadine

Fluoroquinolones Levofloxacin

Moxifloxacin Gemifloxacin Ciprofloxacin

Glycopeptides Vancomycin

Teicoplanin

Lincosamide Clindamycin

Macrolides Azithromycin

Clarithromycin

Nitroimidazole Metronidazole

Oxazolidinone Linezolid

Penicillins Nafcillin

Ampicillin Amoxicillin

Polymyxin E Colistin

Sulfonamides Trimethoprim/Sulfamethoxazole

Tetracyclines

Minocycline Doxycycline Tigecycline

**Table S4.** Summary of clinical data for gut microbiome analyzed 98 patients.

| **Characteristics** | **Non-CRE** | **CRE** | **Overall** | ***P* value** |
| --- | --- | --- | --- | --- |
|  | **(n = 51)** | **(n = 47)** | **(n = 98)** |  |
| Male/Female, n/n | 22/29 | 25/22 | 47/51 | 1.0/1.0 |
| Age (years), mean ± SD | 77.2 ± 9.4 | 72.9 ± 12.5 | 75.2 ± 11.2 | 0.11 |
| Body mass index (kg/m^2^), mean ± SD | 21.8 ± 3.6 | 22.5 ± 3.5 | 22.1 ± 3.6 | 0.22 |
| Co-morbidities, n (%) |  |  |  |  |
| Adrenal Insufficiency | 1 (1.96%) | 1 (2.12%) | 2 | 1.0 |
| Cancer | 8 (15.68%) | 3 (6.38%) | 11 | 0.203 |
| Cerebrovascular accident (CVA) | 25 (49.01%) | 21 (44.68%) | 46 | 0.69 |
| Chronic kidney disease (CKD; eGFR<30) | 7 (13.72%) | 9 (19.14%) | 16 | 0.586 |
| Congestive heart failure (CHF) | 7 (13.72%) | 5 (10.63%) | 12 | 0.761 |
| Coronary artery obstructive disease (CAOD) | 6 (11.76%) | 4 (8.51%) | 10 | 0.742 |
| Dementia | 15 (29.41%) | 8 (17.02%) | 23 | 0.161 |
| Diabetes (DM) | 19 (37.25%) | 18 (38.29%) | 37 | 0.999 |
| Hypertension (HTN) | 29 (56.86%) | 26 (55.31%) | 55 | 0.999 |
| Liver cirrhosis (LC) | 2 (3.92%) | 0 (0.0%) | 2 | 0.495 |
| Pressure ulcer (Sore) | 2 (3.92%) | 9 (19.14%) | 11 | 0.023 |
| Respiratory disease (RD) | 10 (19.6%) | 8 (17.02%) | 18 | 0.798 |
| Surgery, n (%) | 8 (15.68%) | 10 (21.27%) | 18 | 0.603 |
| Treatment, n (%) |  |  |  |  |
| Antibiotics | 38 (74.5%) | 46 (97.87%) | 84 | <0.001 |
| H2 blocker | 7 (13.72%) | 5 (10.63%) | 12 | 0.761 |
| Immunosuppressants | 1 (1.96%) | 2 (4.25%) | 3 | 0.606 |
| Metformin | 6 (11.76%) | 7 (14.89%) | 13 | 0.768 |
| PPI | 19 (37.25%) | 39 (82.97%) | 58 | <0.001 |
| Statin | 15 (29.41%) | 21 (44.68%) | 36 | 0.144 |
| Antibiotics treatment period (days), mean ± SD | 6.4 ± 5.8 | 16.6 ± 8.4 |  | <0.001 |
| PPI treatment period (days), mean ± SD | 4.7 ± 18.3 | 23.5 ± 22.0 |  | <0.001 |
| CRE species detected (n) |  |  |  |  |
| *Enterobacter cloacae* | 0 | 1 | 1 |  |
| *Escherichia coli* | 0 | 3 | 3 |  |

| *Klebsiella oxytoca* | 0 | 3 | 3 |
| --- | --- | --- | --- |
| *Klebsiella pneumoniae* | 0 | 39 | 39 |
| *Serratia marcescens*  CRE genotypes detected (n) | 0 | 2 | 2 |
| Kpc-CPE | - | 39 | 39 |
| Ndm-CPE | - | 3 | 3 |
| Non-typeable CPE | - | 0 | 0 |
| Oxa-CPE | - | 1 | 1 |
| Vim/Imp-CPE | - | 5 | 5 |

CRE, Carbapenem-resistant Enterobacteriaceae; SD, Standard deviation; eGFR, Estimated glomerular filtration rate; PPI, Proton pump inhibitors, CPE, Carbapenem-producing Enterobacteriaceae; Kpc, *Klebsiella pneumoniae* carbapenemase; NDM, New Delhi metallo-β-lactamase; OXA, oxacillinase; VIM, Verona integron metallo-β- lactamase; Imp, imipenemase.

**Table S5.** Odd ratios for clinical variables associated with carbapenem-resistant Enterobacteriaceae (CRE) colonization in gut microbiome analyzed 98 patients.

| **Characteristics Non-CRE CRE** | | | **Mul**  **Odds ratio** | **tivariable analy**  **95% CI** | **sis**  ***P* value** |
| --- | --- | --- | --- | --- | --- |
| Gender (Female) | 29 | 22 | 1.06 | 0.32, 3.55 | 0.927 |
| Co-morbidities  Atrial fibrillation | 1 | 1 | 1.20 | 0.23, 6.33 | 0.83 |
| Cancer | 8 | 3 | 0.20 | 0.03, 1.31 | 0.092 |

Cerebrovascular accident Chronic kidney

| 25 | 21 | 0.57 | 0.16, 2.01 | 0.383 |
| --- | --- | --- | --- | --- |
| 7 | 9 | 1.64 | 0.32, 8.37 | 0.551 |
| 7 | 5 | 0.31 | 0.04, 2.36 | 0.26 |
| 6 | 4 | 0.20 | 0.02, 1.69 | 0.14 |

disease (eGFR<30) Congestive heart failure

Coronary artery

| obstructive disease  Dementia | 15 | 8 | 0.23 | 0.05, 0.99 | 0.048 |
| --- | --- | --- | --- | --- | --- |
| Diabetes | 19 | 18 | 0.56 | 0.12, 2.71 | 0.471 |
| Hypertension | 29 | 26 | 0.60 | 0.17, 2.14 | 0.432 |
| Liver cirrhosis | 2 | 0 | NA | NA, NA | 0.99 |
| Pressure ulcer (Sore) | 2 | 9 | 21.93 | 1.76, 272.33 | 0.016 |
| Respiratory disease | 10 | 8 | 1.53 | 0.28, 8.43 | 0.623 |
| Surgery | 8 | 10 | 1.59 | 0.32, 7.96 | 0.57 |
| Treatment |  |  |  |  |  |
| Antibiotics | 38 | 46 | 14.77 | 0.68, 320.54 | 0.086 |
| H2 blocker | 7 | 5 | 1.56 | 0.24, 10.22 | 0.644 |
| Immunosuppressants | 1 | 2 | 1.03 | 0.03, 36.49 | 0.985 |
| Metformin | 6 | 7 | 1.31 | 0.18, 9.26 | 0.789 |
| PPI | 19 | 39 | 8.93 | 2.13, 37.41 | 0.003 |
| Statin | 15 | 21 | 2.11 | 0.48, 9.37 | 0.325 |

CI, confidence interval; eGFR, Estimated glomerular filtration rate; PPI, Proton pump inhibitors; NA, Not applicable.

**Figure S1.** Significantly different functional features in the gut microbiomes between groups. (A) Heatmap analysis of different features among four groups. (B) Comparison of functional features between paired groups. CRE, carbapenem-resistant Enterobacteriaceae; PPI, proton pump inhibitor; PNT, PPI-not-treated; PT, PPI-treated. ****P* < 0.001, ** *P* < 0.01, * *P* < 0.05.


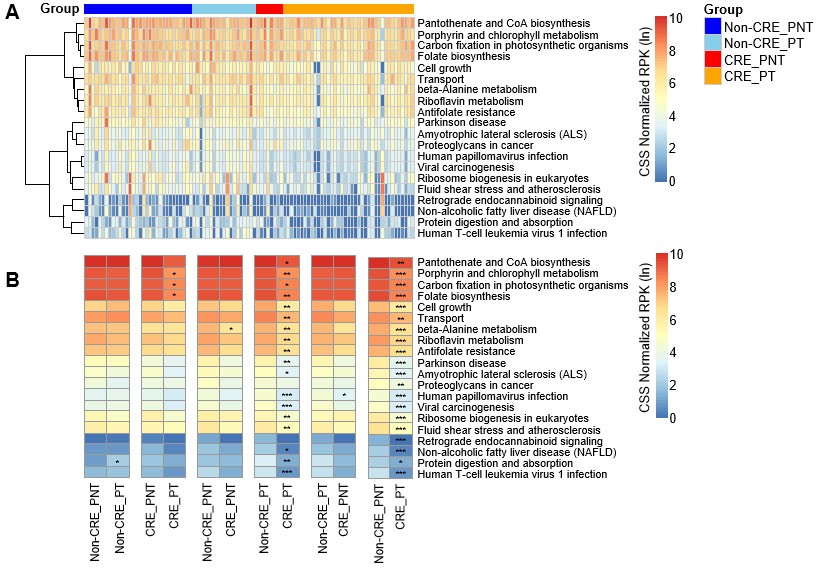


**Figure S2.** Statistics of the constructed metagenome-assembled genomes (MAGs). MAGs with a completeness >50% and redundancy (contamination) <10% were used for analyses. Here, 737 (66.64%) MAGs had a completeness >80% and 566 (51.17%) MAGs had a completeness

>90%; 1,035 (93.58%) MAGs had a redundancy <5%. CRE, carbapenem-resistant Enterobacteriaceae; PPI, proton pump inhibitor; PNT, PPI-not-treated; PT, PPI-treated.


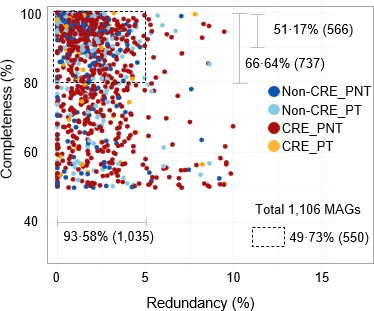


**Figure S3.** Phylogenetic tree of reconstructed metagenome-assembled genomes (MAGs) with statistics. In total, 1,106 MAGs were assigned to seven phyla. The average completeness of the MAGs was 84.16%, and the average redundancy was 1.98%. The inner phylogenetic tree was drawn using the unweighted pair group method with arithmetic mean (UPGMA), and colors indicate different phyla. Outer sections indicate the statistics of each MAG. From the inside to outside, bar charts in the outer sections indicate the completeness (red), redundancy (blue), GC ratio (yellow), N50 (green), and size of MAG (purple). The range of statistics was decided from the smallest to the highest value.


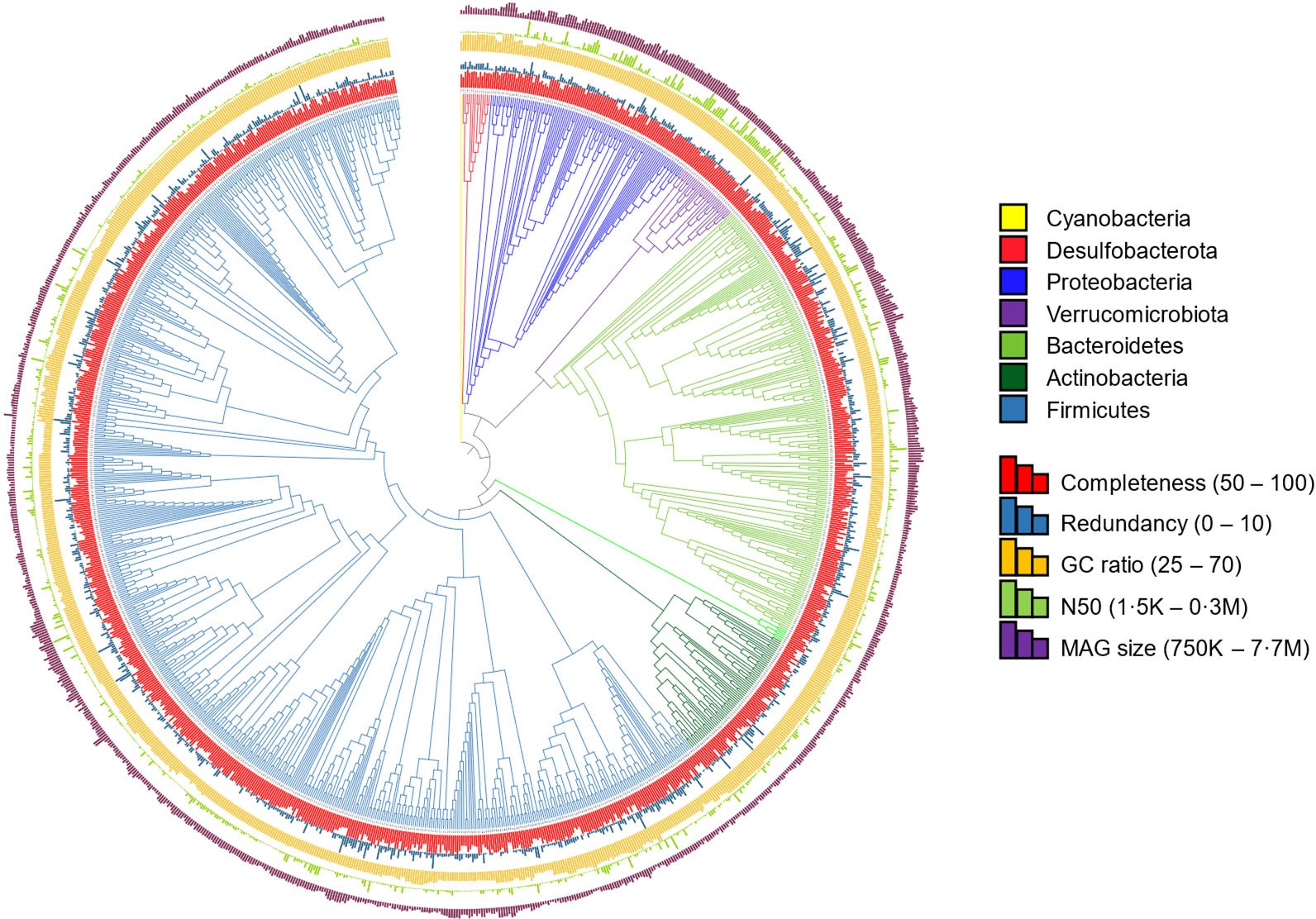


**Figure S4**. Comparison of mobile genetic elements (MGEs) in the gut microbiome among groups. (A) Abundance of MGEs detected from metagenome-assembled genomes (MAGs) in the gut microbiomes were compared between the non-CRE and CRE group according to PPI treatment. Five categories of MGEs, including integration/excision (IE), phage, replication/recombination/repair (RRR), stability/transfer/defense (STD), and transfer, were selected based on the mobileOG-db database. (B) Distribution of MGEs among taxa was analyzed at phylum and family levels. The highest number of MGEs were detected in MAGs of Burkholderiaceae and Enterobacteriaceae families within the Proteobacteria phylum. CRE, carbapenem-resistant Enterobacteriaceae; PPI, proton pump inhibitor; PNT, PPI-not-treated; PT, PPI-treated. *** *P* < 0.001, ** *P* < 0.01, * *P* < 0.05.


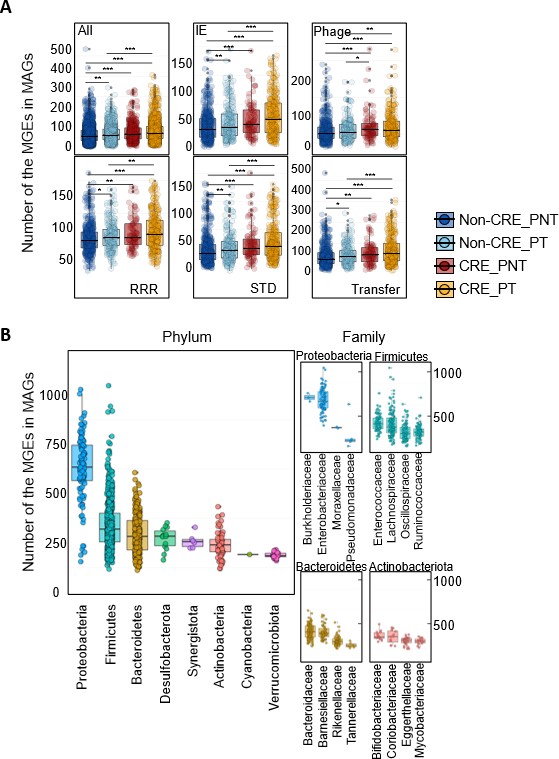

Supplement: Supplementary materials_revision.docx [file KGMI_A_2341635_SM2169.docx]
